# Improved Detection and Monitoring of Fungicide Resistance in *Blumeria graminis* f. sp. *hordei* With High-Throughput Genotype Quantification by Digital PCR

**DOI:** 10.3389/fmicb.2018.00706

**Published:** 2018-04-13

**Authors:** Katherine G. Zulak, Belinda A. Cox, Madeline A. Tucker, Richard P. Oliver, Francisco J. Lopez-Ruiz

**Affiliations:** ^1^The Fungicide Resistance Group, Centre for Crop and Disease Management, School of Molecular and Life Sciences, Curtin University, Bentley, WA, Australia; ^2^Centre for Crop and Disease Management, School of Molecular and Life Sciences, Curtin University, Bentley, WA, Australia

**Keywords:** digital PCR, DMI, fungicide resistance, azole, *Blumeria graminis* f. sp. *hordei*, powdery mildew, CYP51

## Abstract

The increased occurrence of triazole fungicide resistant strains of *Blumeria graminis* f. sp. *hordei* (*Bgh*) is an economic concern for the barley industry in Australia and elsewhere. High levels of resistance to triazoles in the field are caused by two separate point mutations in the *Cyp51* gene, Y136F and S509T. Early detection of these mutations arising in pathogen field populations is important as this allows time for changes in fungicide practices to be adopted, thus mitigating potential yield losses due to fungicide failure and preventing the resistance from becoming dominant. A digital PCR (dPCR) assay has been developed for the detection and quantification of the Y136F and S509T mutations in the *Bgh Cyp51* gene. Mutation levels were quantifiable as low as 0.2% in genomic DNA extractions and field samples. This assay was applied to the high throughput screening of *Bgh* field and bait trial samples from barley growing regions across Australia in the 2015 and 2016 growing seasons and identified the S509T mutation for the first time in the Eastern states of Australia. This is the first report on the use of digital PCR technology for fungicide resistance detection and monitoring in agriculture. Here we describe the potential application of dPCR for the screening of fungicide resistance mutations in a network of specifically designed bait trials. The combination of these two tools constitute an early warning system for the development of fungicide resistance that allows for the timely adjustment of management practices.

## Introduction

The management of fungicide resistance has become a major preoccupation of fungicide manufacturers, regulatory authorities and growers in the last two to three decades. A substantial body of research has evaluated the methods that can be used to prevent or delay the evolution of a resistant population of a pathogen (van den Bosch et al., 2011, 2014). These methods include the withdrawal or substitution of the affected fungicide class with another, mixing or alternating two or more modes of action and the use of genetically resistant crops or rotations. Although there is some controversy over the relative efficacy and practicality of these methods, one aspect is clear; as soon as a resistant population is detected, the fungicide regime must change or the resistant population will increase in frequency. Furthermore, theoretical considerations predict that if a resistant population can be detected early and at low frequencies, the greater is the chance that alterations in the fungicide regime will result in control of the resistant strains.

Fungicide resistance has become particularly problematic for the triazole or demethylase inhibitor (DMI) class of fungicides (Parker et al., 2014), which are critical components of strategies to control diseases of humans, animals (Chamilos and Kontoyiannis, 2005) and plants (Poole and Arnaudin, 2014). We previously reported that the almost ubiquitous planting of susceptible barley varieties and reliance on a single mode of action of fungicide in Western Australia from 2005 resulted in the development of the most damaging and costly epidemic of fungicide resistance ever recorded (Tucker et al., 2015). The use of triazole fungicides in agriculture has also impacted on human health, where mutations that confer resistance to DMIs in clinical *Aspergillus fumigatus* were recently found to be acquired from the general environment (Snelders et al., 2012). Therefore, there is a need for molecular tools that are able to rapidly identify specific mutations conferring fungicide resistance in both clinical and agricultural settings, as early detection of resistance assists adaptive fungicide use practices and effective disease management. Traditionally, methods for identifying resistance in the field involved collecting samples from diseased crops, isolating the fungus in pure culture and conducting fungicide sensitivity bioassays (Ishii and Hollomon, 2015). These phenotyping methods are time consuming, labor intensive and the efficiency of detecting resistance is low because results are based on a limited number of isolates.

Barley powdery mildew is caused by the biotrophic ascomyceteous fungus *Blumeria graminis* f. sp. *hordei* (*Bgh*) and is one of the most common diseases of barley crops worldwide (Jørgensen, 1992). Under optimal environmental conditions, *Bgh* has the potential to reduce barley yields by up to 15–20% and occasionally up to 40% (Chaure et al., 2000). In Western Australia, barley powdery mildew resistance to DMI fungicides has been particularly problematic. The widespread planting of *Bgh* susceptible varieties combined with the reliance on DMIs, most commonly tebuconazole, for *Bgh* control has led to a substantial decrease in efficacy of some DMIs against barley powdery mildew and a growing epidemic of fungicide resistance (Tucker et al., 2015).

Demethylase inhibitor fungicides target lanosterol demethylase, the product of the *Cyp51* gene, and disrupts sterol biosynthesis in cell membranes. Molecular studies of *Bgh* isolates have found that accumulations of single nucleotide mutations in the *Cyp51* gene are correlated with reductions in triazole sensitivity (Wyand and Brown, 2005; Tucker et al., 2015). The first mutation associated with a shift in sensitivity to DMIs was a substitution of a phenylalanine for a tyrosine at position 136 (Y136F corresponds to the archetype *Y137F*; Mair et al., 2016), resulting from a single nucleotide change (Délye et al., 1998). Wyand and Brown (2005) identified that *Bgh* isolates having high levels of resistance to triadimenol also had the Y136F mutation and an additional novel mutation, K147Q. In Western Australia, the Y136F mutation was first identified in *Bgh* isolates in 2009, and has since been found in all *Bgh* samples across Australia, but as the wild type was not found the mutation could not be correlated with tebuconazole failure in the field due to quarantine restrictions. In subsequent years additional isolates were collected and further mutations identified two significant genotypes. A change of serine to threonine at amino acid 509 (S509T corresponds to the archetype *S524T*; Mair et al., 2016), was first detected in 2010 and had become almost universal in Western Australia by 2011. In fact, strains with two mutations in the *Bgh Cyp51* gene, Y136F and S509T, virtually replaced the existing population in just four seasons. The S509T mutation aligned with the S524T change in *Zymoseptoria tritici*, which has been previously characterized as conveying large reductions in triazole sensitivity (Cools and Fraaije, 2013). Fungicide resistance bioassays concluded that *Bgh* isolates with Y136F/S509T genotype were less sensitive to triazoles compared to the Y136F only genotype, with tebuconazole being the most severely compromised (Tucker et al., 2015). A small number of Australian isolates from outside of Western Australia were also characterized, and these were all harboring only the Y136F mutation.

With advances in molecular techniques and the characterization of mutations associated with fungicide resistance, genotyping methods that are able to detect low frequency alleles and avoid the need for pathogen isolation have been implemented successfully. Ideally, a mutation detection system should be sensitive, quantitative, deliver results rapidly and be cost effective. Sanger DNA sequencing can be used to detect single nucleotide polymorphisms (SNPs), however, it can be expensive for multiple samples and there is a time delay to receive results. Allele-specific PCR (Aoki et al., 2011), PCR-cleaved amplified polymorphic sequences (CAPS; Lesemann et al., 2006), and PCR-restriction fragment length polymorphism (PCR-RFLP; Rosenzweig et al., 2014) have all been used for rapid detection of fungicide resistance but are not quantitative. Real-time PCR assays have also been used to quantify fungicide resistance in *B. graminis* f. sp. *tritici* (Fraaije et al., 2002; Yan et al., 2009) as well as *Erysiphe necator* (Dufour et al., 2011); and although quantitative and more sensitive than other PCR-based methods, quantitation relies on standard curves and is often time consuming. Other novel methods include PCR-luminex, which has been developed for the detection of mutations associated with fungicide resistance in the rice blast fungus *Magnaporthe oryzae* (Ishii et al., 2008) but is not quantitative. More recently, loop-mediated isothermal amplification (LAMP) assays have been applied to detect fungicide resistance in *Fusarium* sp. (Duan et al., 2014, 2016) and *Sclerotinia sclerotiorum* (Duan et al., 2015). Although LAMP has the advantage of being a rapid and sensitive method that has the potential for in-field applications, results are not quantitative. Therefore, the challenge is to develop tools that can rapidly detect and quantify a very low abundance of mutant alleles in mixed populations present in field samples with a high enough level of sensitivity to identify single nucleotide changes.

The term ‘digital PCR’ (dPCR) was first used in 1999 (Morley, 2014) and was developed as a quantitative, high-throughput, cost effective method over 20 years ago for leukemia research (Sykes et al., 1992). It is becoming widely used in clinics for rare mutation detection and nucleic acid quantification as it is well suited for these purposes (Huggett et al., 2015; Cao et al., 2017). Digital PCR can be used to accurately quantify nucleic acids with unparalleled sensitivity and has a rapid turnaround time compared to other genotyping methods, with results from samples achieved in 1 day. The reaction chemistry used for dPCR is the same as real-time PCR assays, however, for dPCR the sample is diluted to a point where statistically one or zero molecules occupy a reaction chamber. The reaction is then dispersed into a mass of compartments in which fluorescence values are read individually, eliminating variations due to differences in amplification efficiency and without reliance on standard curves that may vary from experiment to experiment. Based on the Poisson distribution, the number of template copies present in the sample can be calculated from the number of compartments in which amplification has occurred. Several dispersion methods exist for dPCR systems including droplet-, microwell-, channel- and printing-based, each having their own advantages and disadvantages (Cao et al., 2017).

Applications of dPCR for agriculture are emerging, and include screening for genetically modified organisms in seed samples (Fu et al., 2015), studies on populations dynamics of four species of *Aspergillus* infection on grapes (Palumbo et al., 2016), detection of low levels of *Phytophthora nicotianae* in environmental samples (Blaya et al., 2016) and studies of virus transmission and infection on grape vines (Bahder et al., 2016). However, to our knowledge, no studies to date have used dPCR to detect fungicide resistance in field samples of crop plants.

A dPCR assay for the detection and quantification of fungicide resistant genotypes of *Bgh* harboring the Y136F and S509T mutations has been developed and applied to the testing of barley field samples collected from sites across Australia in 2015 and 2016. The use of this technology in screening samples from a network of bait trials serves as an early warning system for the development of fungicide resistance.

## Materials and Methods

### *Bgh* Field Samples and Isolates

In order to evaluate the prevalence of the T509 allele in Western Australia and to determine its presence in other barley growing states, a total of 87 infected barley samples were collected from field trips and/or bait trials deployed across Australia in regions with high barley powdery mildew disease risk; including Western Australia, Victoria, New South Wales, Queensland and Tasmania during the 2015 and 2016 growing seasons (**Table 1** and Supplementary Tables S2, S4, S5). Bait trials sown with the *Bgh* susceptible variety Baudin were designed to increase the frequency of resistant strains. Plots of 2 m × 4 m were sprayed with 1× or 2× the maximum registered dose of one of the fungicides epoxiconazole (0.25 L ha^-1^ and 0.5 L ha^-1^), tebuconazole (0.29 L ha^-1^ and 0.58 L ha^-1^), fluxapyroxad (0.5 L ha^-1^ and 1 L ha^-1^) or azoxystrobin (0.64 L ha^-1^ and 1.28 L ha^-1^), at growth stages GS31 and GS39 (Zadoks et al., 1974). The control plot was sprayed with water. Up to five *Bgh* infected leaf samples were randomly collected per field or experimental plot. While bait trials were used to increase the probability of finding mutant isolates, field samples were used to gauge the prevalence of the mutant isolates in the general fungal population. Field samples were sent to us by growers, crop advisors, the Department for Primary Industries and Regional Development (DPIRD) and the barley powdery mildew outreach project Mildew Mania^1^. Both field trip and bait trial samples were immediately placed in a 15 ml polypropylene tube containing 3 ml 50 mg L^-1^ benzimidazole water agar (Tucker et al., 2013), sealed and transported in coolers with ice packs. Conidia from each sample were transferred to detached barley (*Hordeum vulgare* cv. Baudin) leaves, isolated by single sporing and maintained as previously described by Tucker et al. (2013). The remaining sample material was subjected to DNA extraction and dPCR analysis.

**Table 1:** Origin of *Blumeria graminis* f. sp. *hordei* field samples collected in 2015 and 2016 growing seasons.

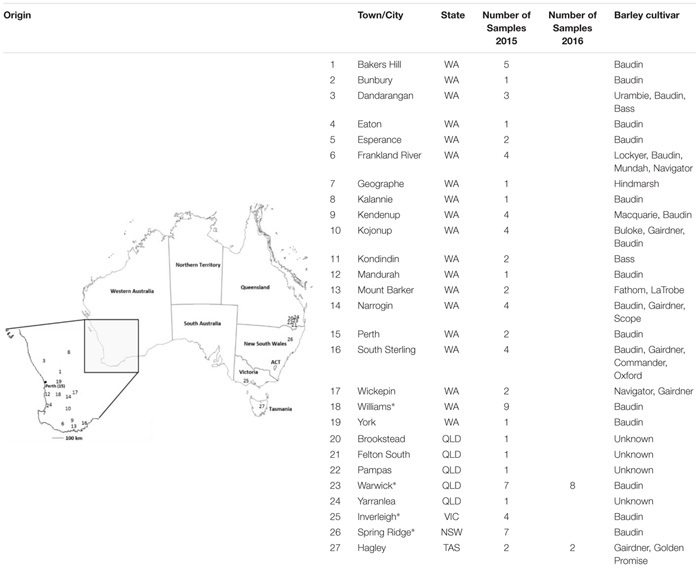

*^∗^Bait trial location. WA, Western Australia; QLD, Queensland; VIC, Victoria; NSW, New South Wales; TAS, Tasmania.*

Previously collected *Bgh* isolates with known *Cyp51* genotypes were used to validate dPCR assays. Per is a triazole resistant isolate (F136/T509) from Western Australia, and Wagga is a triazole sensitive isolate (F136/S509) from New South Wales, Australia (Tucker et al., 2015). DH14 is a triazole sensitive wild type isolate (Y136/S509) from United Kingdom (Spanu et al., 2010). Only genomic DNA was used for DH14 due to quarantine restrictions.

### DNA Extractions

All genomic DNA extractions were carried out using a BioSprint 15 instrument and BioSprint 15 DNA plant kit (Qiagen) according to the manufacturer’s protocol. DNA concentrations were determined using a Quantus fluorometer (Promega) and extractions were stored in sterile double distilled water at -20°C for up to 2 days until dPCR analysis. For *Bgh* field samples, small sections of highly infected barley leaf material were excised with a sterile blade and up to 50 mg leaf material and mycelia were transferred to a 2 ml microfuge tube for total DNA extraction. Where multiple samples were received from one field location, with same cultivar and fungicide spray treatment, subsamples were taken from up to five leaves and pooled together for DNA extraction. An uninfected barley cv. Baudin leaf was included in each set of DNA extractions as a negative control.

For pure single-spored *Bgh* isolates, conidia were dislodged into a dry sterile glass plate and collected in a 1.5 ml microfuge tube using sterile razor blades and subjected to DNA extraction.

### Primers and Dual-Labeled Probes

Digital PCR primers and dual-labeled probes for the Y136F and S509T assays were designed targeting the nucleotide positions in the first and third exons, respectively, of the *Bgh Cyp51* gene according to an alignment of sequences of *Cyp51* from Australian isolates Pshk1 (GenBank accession no. KM016904), Pshk2 (GenBank accession no. KM016905), Strl1 (GenBank accession no. KM016903) and Frnk1 (GenBank accession no. KM016902), and the reference DH14 sequence (GenBank accession no. AJ313157; data not shown). dPCR primers and probes were designed using the OligoArchitect^TM^ software (Sigma-Aldrich). Locked nucleic acid (LNA) modifications were added to the probes to increase their specificity (**Table 2**). NCBI BLAST searches were carried out to check primers and probe sequences were specific to *Bgh Cyp51.* All oligonucleotides and probes were manufactured by Sigma-Aldrich, according to the sequences and modifications given in **Table 2**.

**Table 2:** PCR primers and probes used in this study.

Name	Description	Sequence (5′–3′)	Product size (bp)
509CF	Forward primer – dPCR S509T assay	GTCCCTCTTCTCCCATGCAAT	73
509CR	Reverse primer – dPCR S509T assay	TGCCGAAACGGATTACTCAAG	73
S509 Probe	dPCR probe S509	Calorange560-AGTATG**T**TT**T**CT**C**GG**C**CAA-BHQ1	NA
T509 Probe	dPCR probe T509	FAM-AGTATG**T**TT**A**CT**C**GG**C**CAA-BHQ1	NA
136AF	Forward primer – dPCR Y136F assay	ATGCCGAAGAAATTTATACG	150
136AR	Reverse primer – dPCR Y136 assay	GAACTGTGCAAATATCAGAG	150
Y136 Probe	dPCR probe Y136	FAM-AGGA**C**AG**T**CA**A**AC**A**CTACA-BHQ1	NA
F136 Probe	dPCR probe F136	Calorange560-AGGA**C**AG**T**CA**T**AC**A**CTACA-BHQ1	NA
Bgh51_1F	*Cyp51* gene sequencing primer	TAGACTTCCATTTTCCGTCCT	736
Bgh51_1R	*Cyp51* gene sequencing primer	GGGTGTGTGAAGCAGTGTATATCGT	736
Bgh51_2F	*Cyp51* gene sequencing primer	TATCGATGCAGTAATGGCTGA	700
Bgh51_2R	*Cyp51* gene sequencing primer	AGTGTCCCAACGATGTGGAT	700
Bgh51_3F	*Cyp51* gene sequencing primer	AGTAAAGAATCCAATGCCCGT	528
Bgh51_3R	*Cyp51* gene sequencing primer	CATCAATTGGCAGGTAGTGA	528
BghCYP509F	Amplicon sequencing forward primer	CCACCATGGTTCGCAGTTTC	280
BghCYP509R	Amplicon sequencing reverse primer	TGGTAGCTACGGTCCAGTCA	280

*Nucleotides shown in bold are locked nucleic acids (LNA). BHQ1, Black hole quencher 1.*

For dPCR assays, three sets of primers (A, B, and C) for both the Y136F and S509T dPCR assays were evaluated by standard PCR and dPCR on *Bgh* genomic DNA. For the Y136F and S509T assays, primer pairs A and C, respectively (**Table 2**) generated amplicon bands of the expected size (data not shown) and were selected for subsequent dPCR optimisation.

Primers to sequence the full coding region of the *Bgh Cyp51* gene were designed according to the DH14 reference isolate sequence (**Table 2**). For amplicon sequencing, primers targeting the S509T mutation site in the *Cyp51* gene were developed (**Table 2**).

### Standard PCR

To test the amplification of *Bgh* genomic DNA with dPCR primers, standard PCR was carried out on genomic DNA of pure single-spored *Bgh* isolates. Each 10 μl PCR reaction contained 0.5 U MyTaq DNA polymerase (Bioline) with 1 × MyTaq reaction buffer, 0.4 μM of each primer and 1 μL DNA template. DNA template was serial dilutions of *Bgh* DNA from 100 to 0.1 ng μL^-1^. PCR amplification was performed with the following cycling conditions: initial denaturation 94°C for 2 min, followed by 35 cycles of 94°C for 30 s, 60°C for 30 s, 72°C for 30 s, and a final extension of 72°C for 5 min. PCR products were visualized on a 1% agarose TAE gel stained with SYBR Safe (Thermo Fisher Scientific).

### dPCR Y136F and S509T Assays

Digital PCR was performed on a QuantStudio^TM^ 3D Digital PCR System (Applied Biosystems). Reaction mixtures were prepared by combining 2–5 μL DNA template with QuantStudio^TM^ 3D Digital PCR 2 × Master Mix (Thermo Fisher Scientific), 200 nM of each probe and 900 nM of each primer. 14.5 μL of each reaction mixture was loaded onto a QuantStudio^TM^ 3D Digital PCR Chip and cycled on a Geneamp 9700 flat block thermal cycler (Applied Biosystems) under the following conditions: 96°C for 10 min, then 40 cycles of 58°C or 60°C annealing for 2 min, 98°C for 30 s, followed by 58°C or 60°C for 2 min, then 10°C hold. The annealing temperature was 58°C for the Y136F assay and 60°C for the S509T assay. The chip contained 20,000 partitions with an individual partition volume of 755 pL. End-point fluorescence data were collected and analyzed using the QuantStudio^TM^ 3D Digital PCR Instrument and 3D AnalysisSuite^TM^ software, version 3.0. Data was manually edited to remove outlying data points that were not located within the clusters on the scatter plots.

For dPCR assays, 0.5 ng *Bgh* genomic DNA per reaction was used to achieve the optimal recommended concentration range of 200–2000 copies μl^-1^ for quantification (QuantStudio^TM^ 3D Digital PCR System User Guide 2015). For *Bgh*, *Puccinia hordei*, *Pyrenophora teres* f. sp. *teres*, and *P. teres* f. sp. *maculata* infected leaf samples, individual barley leaves were infected using a detached leaf assay as described in Tucker et al. (2013). For subsequent DNA extractions, 2–5 μL of total DNA extraction was used per dPCR reaction, depending on the amount of *Bgh* present on the leaves. If the dPCR chip result was outside the range of 200–2000 copies μL^-1^, the reaction was repeated with diluted DNA, or more DNA template where possible.

In order to evaluate the specificity of the Y136F and S509T assays, dPCR was performed on genomic DNA samples of pure *Bgh* isolates Per, Wagga and DH14 with known genotypes F136/T509, F136/S509, and Y136/S509, respectively.

For assays containing known ratios of genomic DNA, extractions of *Bgh* isolates Per and Wagga were quantified and adjusted to equal DNA concentrations with water. Per was serially diluted in Wagga genomic DNA in ratios as follows: 90, 75, 50, 25, 10, 5, 1, 0.1, and 0.01%. Chips were run in triplicate for each dilution. Further optimization of the Y136F assay was deemed unnecessary because every isolate tested contained this allele.

In order to evaluate the assay for the detection of the T509 mutation on field samples, we inoculated leaves of a *Bgh*-susceptible barley cultivar Baudin with isolates Per and Wagga. To simulate field conditions and to test for interspecies cross reaction, Baudin leaves were inoculated with common barley pathogens *Puccinia hordei*, *Pyrenophora teres* f. sp. *teres*, and *Pyrenophora teres* f. sp. *maculata*.

Statistical analysis was performed using IBM SPSS. Comparison of mean percent mutant among Western Australia, Queensland, Victoria, and New South Wales was performed using a one way ANOVA with significance set at 0.05. Comparison between samples from Queensland collected in 2015 and 2016 was performed using an independent *T*-test with significance set at 0.05.

### Sequencing of *Bgh Cyp51* Gene

In order to verify dPCR results from field samples, the complete *Cyp51* gene sequences were obtained for eight *Bgh* single spored isolates produced from field samples along with the triazole resistant isolate Per and the sensitive isolate Wagga. Sequences were obtained by Sanger sequencing (Macrogen, Seoul, South Korea) and aligned in Geneious v 6.1 (Biomatters) with isolates Pshk2 and Frnk 1.

For amplicon sequencing, total DNA from the *Bgh* field sample Queensland 5 was amplified by standard PCR with primers BghCYP509F and BghCYP509R (**Table 2**). The resulting PCR product was purified by QIAquick PCR Purification Kit (Qiagen) and quantified on a Nanodrop (Thermo Fisher). A total of 500 ng PCR product was directly used to construct paired-end libraries using TruSeq Custom Amplicon v1.5 Library Prep Kit and sequenced on an Illumina HiSeq 2000 instrument with PE150 sequencing by Novogene Institute (Beijing, China). Percentage of *Cyp51* T509 was calculated using the ±10 bp region around the mutation site.

## Results

### dPCR Successfully Identifies Y136F and S509T Mutations From Genomic DNA and Infected Leaves

In assays using genomic DNA of pure isolates with known genotypes, dPCR correctly identified the DMI-sensitive isolate Wagga (0.01% T509) and the resistant isolate Per (99.98% T509; **Table 3**). Both resistant and sensitive isolates contain the Y136F mutation and were also correctly identified and quantified as 99–100% F136 (**Table 3**). The reference isolate DH14 does not contain the Y136F or the S509T mutation (Wyand and Brown, 2005) and was correctly identified in both Y136F (0.13% F136) and S509T assays (0.15% T509; **Table 3**). The no template control did not detect either allele.

**Table 3:** Specificity of detection of S509T and Y136F digital PCR assays on genomic DNA of *Blumeria graminis* f. sp. *hordei* isolates.

DNA sample	% T509	CI % T509	Copies μL^-1^ S509	CI Copies μL^-1^ S509	Copies μL^-1^ T509	CI Copies μL^-1^ T509
Per^a^	99.98	95.442–104.55	0.32	0.13–0.76	1863.60	1830.4–1897.5
Wagga^b^	0.01	1.21E-3–4.11E-2	946.68	927.23–966.54	0.07	9.66E-3–0.487
DH14^b^	0.15	8.82E-2–0.253	818.65	799.33–838.44	1.231	0.729–2.078
Water	NA	NA	6.49E-2	9.14E-3–0.416	6.49E-2	9.14E-3–0.461

**DNA sample**	**% F136**	**CI %F136**	**Copies μL^-1^ F136**	**CI Copies μL^-1^ F136**	**Copies μL^-1^ Y136**	**CI Copies μL^-1^ Y136**

Per	99.9	95.54-104.55	489.73	476.95–502.86	7.16E-02	1.01E-2–0.508
Wagga	100	NA	873.63	856.09–891.52	0	NA
DH14	0.13	5.81E-2-0.28	0.454	0.204–1.01	353.68	342.98–364.71
Water	NA	NA	0.07	9.70E-3–0.489	0	NA

*^a^Triazole resistant isolate. ^b^Triazole sensitive isolate. CI = 95% confidence interval.*

**FIGURE 1:**
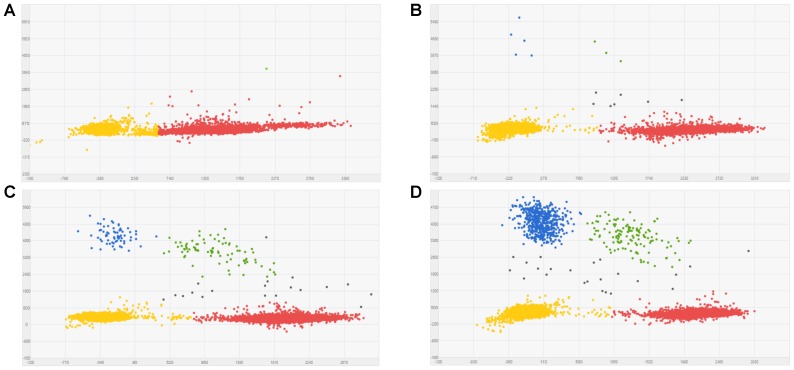
Scatter plots for *Blumeria graminis* f. sp. *hordei* (*Bgh*) S509T digital PCR assay using known ratios of genomic DNA for the triazole resistant *Bgh* isolate Per (contains T509 allele) and triazole sensitive *Bgh* isolate Wagga (contains S509 allele). Scatter plots were obtained by Quantstudio^TM^ 3D AnalysisSuite^TM^ by digital PCR. Wells with T509 alleles are represented by FAM signals (blue), S509 alleles are represented by VIC signals (red), detection of both alleles are represented by green signals, and wells without any alleles (passive reference) are represented by ROX signals (yellow). **(A)** 100% Wagga gDNA (S509 allele only). **(B)** 0.1% Per gDNA; T509 frequency calculated by dPCR = 0.127%. **(C)** 1% Per gDNA; T509 dPCR frequency = 1.06%. **(D)** 10% Per gDNA; T509 dPCR frequency = 12.59%.

**FIGURE 2:**
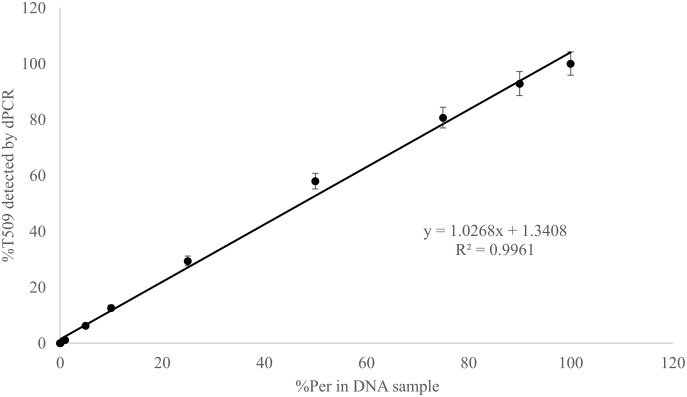
Linear correlation between percent T509 allele in *Blumeria graminis* f. sp. *hordei* genomic DNA samples quantified by digital PCR and the percentage of the triazole resistant isolate Per (T509) in a genomic DNA mixture of Per and the triazole sensitive isolate Wagga (S509). Each point represents the average of triplicate chips.

Known mixtures of Per and Wagga DNA were subjected to dPCR and when viewed as a scatterplot, individual wells fall into discrete groups of either S509 allele only, T509 only, both S509 and T509 and neither allele present (**Figure 1**). When percentage of T509 was plotted against known ratios, the two values correlated well with an *R*^2^ value of 0.996 and results from triplicate chips were consistent (**Figure 2**). The lower level of quantification of the T509 allele was determined to be 0.2%, as the %T509 level for 0.01% Per was similar to that of 100% Wagga and the %T509 and %F136 values for the wild type DH14 isolate were 0.15 and 0.13, respectively, thus indicating a false positive (**Tables 3**, **4**).

**Table 4:** Sensitivity of detection of S509T digital PCR assay on mixtures of genomic DNA of *Blumeria graminis* f. sp. *hordei* isolates.

DNA Sample	% T509	CI % T509	Copies μL^-1^ S509	CI Copies μL^-1^ S509	Copies μL^-1^ T509	CI Copies μL^-1^ T509
100% Wagga^a^	2.74E-02	7.33E-3–9.99E-2	591.48	576.4–606.96	0.162	4.05E-2–0.647
0.01% Per^b^	7.40E-02	2.48E-2–0.218	342.57	331.46–354.05	0.254	8.18E-2–0.787
0.1% Per	0.127	6.63E-2–0.243	513.43	500.3–526.9	0.655	0.341–1.259
1% Per	1.06	0.775–1.455	277.7	268.43–287.29	2.985	2.19–4.07
5% Per	5.92	5.31–6.579	485.32	472.49–498.49	30.511	27.675–33.636
10% Per	12.59	11.61–13.631	456.49	443.83–469.52	65.739	61.385–70.401
25% Per	29.62	27.906–31.396	388.44	376.93–400.3	163.44	156.36–170.85
50% Per	58.44	55.719–61.226	288.57	278.89–298.59	405.75	393.96–417.89
75% Per	80.58	76.84–84.403	130.61	124.08–137.48	541.86	527.21–556.91
90% Per	93.01	89.006–97.08	67.129	62.617–71.967	893.36	873.39–913.78
100% Per	99.94	95.893–104.06	0.451	0.203–1.005	776.3	758.95–794.03
Water	NA	NA	0	NA	0	NA

*^a^Triazole sensitive isolate. ^b^Triazole resistant isolate. CI = 95% confidence interval.*

Digital PCR also correctly identified both the T509 and S509 genotypes from total DNA extracted from barley leaves infected with Per (99.98% T509) and Wagga (0% T509) (Supplementary Table S1). No cross reaction was found in any of the other barley pathogens tested and neither mutation was detected in the no template control (Supplementary Table S1).

### dPCR Successfully Identifies Y136F and S509T Mutations in Field Trip and Bait Trial Samples

**FIGURE 3:**
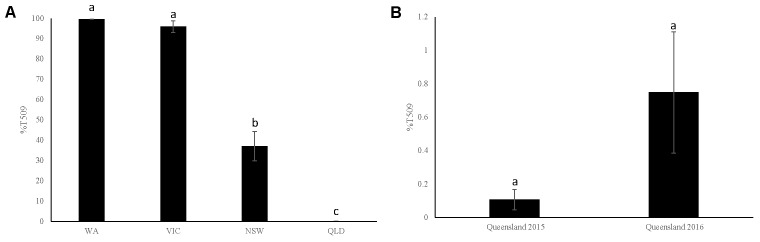
Percentage S509T mutation in field collected barley (*Hordeum vulgare*) leaf samples from across Australia infected with *Blumeria graminis* f. sp. *hordei* as quantified by digital PCR during the 2015 and 2016 growing seasons. **(A)** Average percentage T509 allele in field samples across different states in Australia in 2015. **(B)** Average percentage T509 allele in field samples collected from Queensland during the 2015 and 2016 growing seasons. WA, Western Australia; VIC, Victoria; NSW, New South Wales; QLD, Queensland. Letters above bars indicate statistical significance.

In Western Australia dPCR S509T analysis of field trip samples collected during the 2015 growing season showed an extremely high incidence of this mutation, with an average of 98.74% T509 allele in the population (range 65.76–100%; Supplementary Table S5). Similar results were obtained when samples collected from a 2015 Western Australian bait trial were analyzed (T509 range 99.29–100%, mean T509 = 99.6%; **Figure 3A** and Supplementary Table S2).

Digital PCR S509T analysis of 2015 *Bgh* samples revealed the occurrence of T509 for the first time in Victoria, New South Wales, Queensland, and Tasmania (**Figures 3A,B** and Supplementary Tables S2, S4, S5). The analysis of two field trip samples collected from Tasmania in 2015 (T509 = 84.49 and 99.93%, mean T509 = 92.21%) and two more in 2016 (T509 = 42.04 and 57.21%, mean T509 = 49.62%), confirmed the existence of the mutation in the region (Supplementary Table S5). The analysis of samples collected from bait trials deployed in Victoria and New South Wales showed T509 average levels of 95.97% (range 87.9–100%) and 37.11% (range 7.21–67.68%), respectively (**Figure 3A** and Supplementary Table S2). There was no statistically significant difference between samples from Western Australia and Victoria, however, New South Wales and Queensland were significantly different (**Figure 3A**). Because bait trials enrich for the mutant isolates, these values cannot be used to gauge prevalence in the field.

Although several of the samples collected in Queensland had T509 allele levels above the established 0.2% quantification threshold, T509 copies μL^-1^ were very low compared with the values obtained from samples collected from other states (**Figure 3B** and Supplementary Tables S2, S4, S5). The comparison between the 2015 and 2016 bait trials found no statistically significant differences (*p* = 0.114) in T509 levels with averages of 0.11% (range 0–0.42%) and 0.75% (range 0.13–3.25%), respectively (**Figure 3B** and Supplementary Tables S2, S4).

Digital PCR Y136F analysis was also carried out on 2015 bait trials samples from Victoria, New South Wales, and Queensland. Results revealed that all populations were F136, with levels of 99.9–100% (Supplementary Table S3).

### Genotype Detection Using dPCR Correlates With Sequencing Analysis of Field Samples

*Cyp51* sequences obtained from three isolates from 2015 field samples from Western Australia (Esperance 2, T509 = 100%; Bunbury 1, T509 = 99.97%; and Eaton 1, T509 = 99.95%), one isolate from Victoria (Victoria 1, T509 = 99.62%), one isolate from New South Wales (New South Wales 6, T509 = 67.68%) were all found to be 100% identical to Per (F136/T509) and with 100% sequence identity to the *Cyp51* gene from isolate Pshk2.

*Cyp51* sequences obtained from two isolates from 2015 field and bait trial samples from Queensland (Queensland 7, T509 = 0.14% and Queensland 81, T509 = 0%) were 100% identical to isolate Wagga (F136/S509), with 100% sequence identity to isolate Frnk 1.

In order to determine if the dPCR result for 2015 bait trial sample Queensland 5 of T509 = 0.42% was a real detection of low level mutation or just a false positive result of the dPCR assay, amplicon sequencing was carried out. A total of 4.4 million clean reads from genomic DNA from the field sample Queensland 5 were achieved with 3,930,113 reads containing the S509 allele and 27,345 sequences identified as containing the T509 mutation. The calculated frequency of T509 mutation using amplicon sequencing was 0.69% which is comparable to the 0.49% frequency calculated from dPCR and supports the accuracy of dPCR detection of low level mutation frequency.

## Discussion

In this study, we determined that dPCR can accurately and sensitively measure the level of two mutations in the *Cyp51* gene that confer resistance to DMI fungicides (Y136F and S509T) in *Bgh* samples collected from the field.

Our system of dPCR combined with baiting trials is an especially powerful tool for two reasons: (i) the use of baiting trials allows for enrichment of the mutant population and dPCR can quantify mutations down to 0.2%; thus allowing us to identify cases of resistance quickly with unprecedented sensitivity and accuracy; and (ii) although baiting trials do not portray an accurate measure of mutation field frequency, these results can quickly pinpoint resistance hot spots and allow us to target field sampling, thus saving on time and resources. To our knowledge, this is the first study which uses dPCR to quantify fungicide resistance in the field.

Traditionally, methods for detecting DMI fungicide resistance in *Bgh* involved isolating individual conidia from infected lesions collected from the field and using Sanger sequencing to identify mutations in the target *Cyp51* gene (Wyand and Brown, 2005). This method is time consuming and does not provide an accurate determination of the frequency of mutant isolates within a field sample and will likely miss resistant isolates if they are present at very low levels. In order to overcome this limitation, there have been several studies which use qPCR assays to detect fungicide resistance in field samples of various powdery mildew species. Fungicide resistance to strobilurins (QoI) was profiled in wheat powdery mildew (*B. graminis* f. sp. *tritici*) using a Taqman qPCR assay to detect the G143A mutation within the cytochrome *b* gene, which confers complete resistance to strobilurin fungicides. The limit of detection of the assay was stated to be at least 1 in 10 000 (Fraaije et al., 2002). qPCR was also used to profile resistance to DMIs in wheat powdery mildew, specifically to detect the Y136F mutation in *Cyp51*, however, lower detection and quantification limits are unclear (Yan et al., 2009). In powdery mildew of grape (*E. necator*), a qPCR assay was developed to detect and profile DMI and QoI fungicide resistance in field populations from French vineyards (Dufour et al., 2011). A qPCR assay was designed to identify and quantify a mutation used to distinguish A and B genetic groups and the Y136F mutation that reduces sensitivity to DMI fungicides. The Y136F assay was found to be easier, had a low limit of detection (limit of detection = 0.85%) and was quantifiable (limit of quantification = 2.85%), unlike the more traditionally used CAPS method.

The dPCR assays we have developed share many of the same advantages as qPCR such as a lower detection limit, speed, precision and higher throughput than more traditional single-spore isolation and PCR-based methods. However, digital PCR does present several additional advantages over qPCR based methods. For example, dPCR does not rely on standard curves for quantitation, which both saves time and increases reproducibility between and within laboratories. Additionally, the results from our optimisation experiments and amplicon sequencing suggest that the S509T and Y136F dPCR assays we have developed are sensitive, accurate and robust enough for the early detection of low abundance alleles in infected field samples to a detection limit of 0.2%. This is important because sensitivity and accuracy in the detection of resistance are critical factors to the prevention of potential fungicide resistance outbreaks by allowing for the adjustment of spray programs at the earliest possible opportunity.

Triazole resistance in *Bgh* was first detected in the United Kingdom in 1981 (Fletcher and Wolfe, 1981; FRAC, 2013) but did not significantly develop there due to use of alternative fungicide modes of action and resistant crop varieties. In Western Australia, repeated sowing of *Bgh*-susceptible varieties combined with the widespread, consistent and exclusive use of triazoles over the years resulted in the loss of efficacy. This was not always the case, however, with wild type *Bgh* isolates containing the S509 allele commonly found in Australia prior to 2012 (Tucker et al., 2015). Interestingly, our analyses indicate that there has been a generalized replacement of this allele by its mutated form T509 (Supplementary Tables S2, S4, S5), which has been probably fueled by a strong selection pressure imposed by widespread triazole use.

Unlike Western Australia where there are generalized reports of the lack of performance of some triazoles (especially tebuconazole) for the control of *Bgh*, no resistance reports have been received from any of the other barley growing states. The lack of favorable disease conditions (use of resistant varieties, conducive weather, stubble management, etc.) and/or the existence of an important level of S509 allele in the population, are probably contributing to mitigate the problem. However, the high levels of T509 found in baiting trial samples in Victoria and, to a lesser extent, in New South Wales (**Figure 3A**) suggest that sudden changes in *Bgh* incidence could threaten disease control and quickly lead to a similar scenario to that recorded in Western Australia in recent years (Tucker et al., 2015). These results are concerning and require targeted field sampling to determine the level of DMI resistance in the general *Bgh* field population. The frequency levels of T509 in Queensland baiting trials are relatively low (**Figures 3A,B**) but this mutation was found in both 2015 and 2016 suggesting a sustained presence of a DMI resistant population in Queensland.

We have undertaken extensive field sampling in Western Australia, which have returned consistently high T509 levels (average = 98.74%; Supplementary Table S5), supporting the reports of field failure of DMI fungicides. We have fewer field samples from other states due to distance, however, the four samples we have received from Queensland suggest a low level of DMI resistant *Bgh* isolates in the field (Supplementary Table S5). In contrast, we have received two samples from Tasmania for both 2015 and 2016 that show a relatively higher frequency of the T509 mutation although clearly more sampling is required to gauge what the true field frequency of the T509 mutation (Supplementary Table S5). However, our results do confirm the presence of the mutation in Tasmania.

It would be advantageous to use our combination of dPCR, a nationwide network of regularly sampled baiting trials and targeted field sampling to detect and monitor the evolution of fungicide resistance over time and in response to changing fungicide spray regimes. Our system would also be used to track the amount of time from the first detection of the mutation to changes in fungicide efficacy in the field, as this information is critical for the formulation of evidence-driven resistance management strategies. dPCR is also versatile and can be applied to the detection and monitoring of any number of mutations in various target genes of interest that may confer resistance to classes of fungicides other than DMIs. Thus, if other modes of action are being used in the field, it is possible to easily test for mutational hot spots in several target genes from the same field sample.

Results of our dPCR screening of *Bgh* samples from baiting trials highlights the power of this methodology as an early warning system for fungicide resistance development. dPCR has shown to be a fast, accurate, sensitive and reproducible technology for the screening of point mutations associated with fungicide resistance. Early identification of resistance through the yearly screening of baiting trials followed by targeted field sampling can then be used to adjust spray timing, dosage and mode of action usage with the aim of extending the life of effective fungicides and preventing future fungicide resistance outbreaks.

## Author Contributions

FL-R designed the project. KZ, BC, MT, and RO contributed to the conception of the work. KZ and BC performed the analysis and interpretation of data. KZ, BC, RO, MT, and FL-R wrote and edited the manuscript.

## Conflict of Interest Statement

The authors declare that the research was conducted in the absence of any commercial or financial relationships that could be construed as a potential conflict of interest.

## Footnotes

1 http://mildewmania.com.au/
