# Supplementary material for: Improved Detection and Monitoring of Fungicide Resistance in Blumeria graminis f. sp. hordei With High-Throughput Genotype Quantification by Digital PCR
Source: Front Microbiol. 2018 Apr 13;9:706. doi: 10.3389/fmicb.2018.00706 (PMC5908980; doi:10.3389/fmicb.2018.00706)
Supplement: Supplementary file 1 [file Table_1.docx]

Supplementary Material

Improved detection and monitoring of fungicide resistance in *Blumeria graminis* f. sp. *hordei* with high throughput genotype quantification by digital PCR

Katherine G. Zulak^1^, Belinda Cox^1^, Madeline A. Tucker^1^ Richard P. Oliver^1^ and Francisco Lopez-Ruiz^1*^

*** Correspondence:**Francisco J. Lopez-Ruiz
[fran.lopezruiz@curtin.edu.au](mailto:fran.lopezruiz@curtin.edu.au)

# Supplementary Tables

**Table S1.** Specificity of detection of S509T digital PCR assay on barley leaves (*Hordeum vulgare* cv. Baudin) infected with *Blumeria graminis* f. sp. *hordei* isolates

| DNA sample | % T509 | CI %T509 | Copies µL^-1^ S509 | CI Copies µL^-1^ S509 | Copies µL^-1^ T509 | CI Copies µL^-1^ T509 |
| --- | --- | --- | --- | --- | --- | --- |
| Per^a^ infected leaf | 99.98 | 95.778 -- 104.2 | 0.371 | 0.167 -- 0.825 | 1568 | 1540.1 -- 1596.4 |
| Wagga^b^ infected leaf | 4.66E-03 | 7.71E-4 -- 2.64E-2 | 1330 | 1305.9 -- 1354.6 | 6.20E-02 | 8.73E-3 -- 0.44 |
| *Puccinia hordei* | 0 | NA | 0.396 | 0.178 -- 0.881 | 0.396 | 0.178 -- 0.881 |
| *Pyrenophora teres*  f*.* sp. *teres* | 0 | NA | 6.29E-02 | 8.86E-3 -- 0.447 | 6.29E-02 | 8.86E-3 -- 0.447 |
| *Pyrenophora teres*  f*.* sp. *maculata* | 0 | NA | 6.50E-02 | 9.16E-3 -- 0.461 | 6.50E-02 | 9.16E-3 -- 0.461 |
| Uninfected barley leaf | 0 | NA | 0.268 | 0.101 -- 0.715 | 0.201 | 6.49E-2 -- 0.624 |
| Water | 0 | NA | 0.07 | 9.70E-3-0.489 | 0 | NA |

^a^Triazole resistant isolate

^b^Triazole sensitive isolate

CI = 95% confidence interval

**Table S2.** Digital PCR detection of T509 allele in field samples from bait trials in 2015

| Location | %T509 | CI %T509 | S509 copies µL^-1^ | CI 509 copies µL^-1^ | T509 copies µL^-1^ | CI copies µL^-1^ T509 |
| --- | --- | --- | --- | --- | --- | --- |
| Western Australia 1 | 99.77 | 94.827 -- 104.73 | 5.432 | 4.298 -- 6.866 | 2326.4 | 2283.4 -- 2370.3 |
| Western Australia 2 | 99.35 | 94.021 -- 104.9 | 1.906 | 1.266 -- 2.868 | 292.8 | 282.77 -- 303.19 |
| Western Australia 3 | 99.88 | 94.847 -- 104.93 | 2.831 | 2.023 -- 3.962 | 2254.8 | 2211.6 -- 2298.8 |
| Western Australia 4 | 99.62 | 95.324 -- 103.95 | 5.98 | 4.769 -- 7.499 | 1547.9 | 1518.2 -- 1578.1 |
| Western Australia 5 | 99.70 | 95.254-- 104.25 | 1.572 | 1.014 -- 2.437 | 513.03 | 499.46 -- 526.96 |
| Western Australia 6 | 99.29 | 94.663 -- 104.02 | 4.277 | 3.213 -- 5.692 | 593.89 | 577.93 -- 610.28 |
| Western Australia 7 | 99.50 | 95.33 -- 103.72 | 6.159 | 4.919 -- 7.712 | 1230.6 | 1205.7 -- 1256 |
| Western Australia 8 | 100.00 | 94.969 -- 105.05 | 8.50E-02 | 1.20E-2 -- 0.603 | 2222.2 | 2179.2 -- 2266 |
| Western Australia 9 | 99.32 | 95.099 -- 103.62 | 5.56 | 4.376 -- 7.064 | 815.57 | 796.86 -- 834.73 |
| Victoria 1 | 99.62 | 95.174 -- 104.18 | 2.148 | 1.462 -- 3.154 | 568.95 | 554.13 -- 584.16 |
| Victoria 2 | 87.90 | 83.123 -- 92.877 | 35.912 | 32.725 -- 39.409 | 260.96 | 251.73 -- 270.52 |
| Victoria 4 | 96.39 | 90.951 -- 102.07 | 8.911 | 7.404 -- 10.724 | 237.97 | 229.22 -- 247.05 |
| Victoria 7 | 100 | NA | 0 | NA | 95.532 | 90.159 -- 101.22 |
| New South Wales 1 | 21.14 | 19.493 -- 22.898 | 266.51 | 257.04 -- 276.32 | 71.439 | 66.791 -- 76.411 |
| New South Wales 2 | 45.34 | 42.515 -- 48.303 | 169.1 | 161.89 -- 176.62 | 140.25 | 133.74 -- 147.08 |
| New South Wales 3 | 39.09 | 36.79 -- 41.489 | 241.84 | 233.17 -- 250.83 | 155.19 | 148.39 -- 162.31 |
| New South Wales 4 | 7.21 | 5.3 -- 9.792 | 42.705 | 39.304 -- 46.4 | 3.318 | 2.469 -- 4.458 |
| New South Wales 5 | 41.25 | 38.272 -- 44.407 | 143.1 | 136.3 -- 150.23 | 100.46 | 94.835 -- 106.41 |
| New South Wales 6 | 67.68 | 64.637 -- 70.789 | 266.6 | 257.19 -- 276.35 | 558.3 | 543.81 -- 573.18 |
| New South Wales 7 | 38.06 | 33.273 -- 43.492 | 39.972 | 36.618 -- 43.634 | 24.56 | 21.969 -- 27.457 |
| Queensland 1 | 0 | NA | 1910.8 | 1875 -- 1947.3 | 0 | NA |
| Queensland 2 | 0 | NA | 286.51 | 276.73 -- 296.64 | 0 | NA |
| Queensland 3 | 0 | NA | 1471.4 | 1443.5 -- 1499.7 | 0 | NA |
| Queensland 4 | 0.19 | 0.118 -- 0.294 | 818.17 | 799.64 -- 837.12 | 1.535 | 0.979 -- 2.407 |
| Queensland 5 | 0.42 | 0.375 -- 0.546 | 1070.9 | 1048.7 -- 1093.5 | 4.532 | 3.496 -- 5.876 |
| Queensland 6 | 0 | NA | 1835.9 | 1799.4 -- 1873.1 | 0 | NA |
| Queensland 7 | 0.14 | 9.46E-2 -- 0.209 | 1640.1 | 1607.1 -- 1673.8 | 2.324 | 1.582 -- 3.414 |

CI = 95% confidence interval

**Table S3.** Digital PCR detection of F136 alleles in field samples from bait trials in 2015

| Location | % F136 | CI %F136 | Y136 copies µL^-1^ | CI Y136 copies µL^-1^ | F136 copies µL^-1^ | CI F136 copies µL^-1^ |
| --- | --- | --- | --- | --- | --- | --- |
| New South Wales 1 | 100 | NA | 0 | NA | 384.03 | 372.44 -- 397.77 |
| New South Wales 2 | 100 | NA | 0 | NA | 337.5 | 327.58 -- 349.35 |
| New South Wales 3 | 100 | NA | 0 | NA | 517.68 | 506.57 -- 534.14 |
| New South Wales 5 | 100 | NA | 0 | NA | 181.48 | 174.38 -- 190.37 |
| New South Wales 6 | 100 | NA | 0 | NA | 772.54 | 759.51 -- 794.61 |
| New South Wales 7 | 100 | NA | 0 | NA | 53.198 | 49.333 -- 57.396 |
| Victoria 1 | 100 | NA | 0 | NA | 481.15 | 470.55 -- 497.45 |
| Victoria 2 | 99.91 | 93.726 -- 106.43 | 0.15 | 3.76E-2 -- 0.601 | 172.75 | 165.79 -- 180.42 |
| Victoria 4 | 99.96 | 93.893 -- 106.34 | 7.49E-02 | 1.06E-2 -- 0.532 | 181.47 | 174.23 -- 189.24 |
| Victoria 7 | 100 | NA | 0 | NA | 50.177 | 46.445 -- 54.246 |
| Queensland 1 | 100 | NA | 0 | NA | 1621.4 | 1608.1 -- 1670.3 |
| Queensland 2 | 100 | NA | 0 | NA | 197.14 | 189.51 -- 205.33 |
| Queensland 3 | 100 | NA | 0 | NA | 962.3 | 944.57 -- 989.96 |
| Queensland 4 | 100 | NA | 0 | NA | 572.8 | 560.19 -- 590.19 |
| Queensland 5 | 100 | NA | 0 | NA | 778.07 | 765.06 -- 800.47 |
| Queensland 6 | 100 | NA | 0 | NA | 1677.1 | 1657.9 -- 1720.6 |
| Queensland 7 | 99.99 | 95.698 -- 104.32 | 0.161 | 4.03E-2 -- 0.644 | 1479.4 | 1465.6 -- 1524.8 |

CI = 95% confidence interval

**Table S4.** Digital PCR detection of T509 allele in field samples from bait trials in 2016

| Location | % T509 | CI % T509 | Copies µL^-1^ T509 | CI T509 copies µL^-1^ | Copies µL^-1^ S509 | CI S509 copies µL^-1^ |
| --- | --- | --- | --- | --- | --- | --- |
| Queensland 1 | 0.13 | 7.04E-2 -- 0.219 | 828.46 | 808.86 -- 848.53 | 1.036 | 0.589 -- 1.825 |
| Queensland 2 | 3.25 | 2.556 -- 4.12 | 199.32 | 190.730 – 208.29 | 6.692 | 5.312 – 8.431 |
| Queensland 3 | 0.41 | 0.29 -- 0.587 | 627.93 | 612.19 -- 644.06 | 2.606 | 1.843 -- 3.685 |
| Queensland 4 | 0.65 | 0.384 -- 1.095 | 175.54 | 168.01 -- 183.42 | 1.148 | 0.68 -- 1.939 |
| Queensland 5 | 0.19 | 9.20E-2 -- 0.397 | 299.23 | 289.08 -- 309.73 | 0.575 | 0.274 -- 1.206 |
| Queensland 6 | 0.35 | 0.233 -- 0.533 | 521.48 | 507.57 -- 535.78 | 1.851 | 1.23 -- 2.785 |
| Queensland 7 | 0.54 | 0.332 -- 0.888 | 291.63 | 280.64 -- 303.05 | 1.596 | 0.978 -- 2.605 |
| Queensland 8 | 0.47 | 0.302 -- 0.731 | 367.16 | 355.44 -- 379.28 | 1.738 | 1.121 -- 2.694 |

CI = 95% confidence interval

**Table S5.** Digital PCR detection of T509 allele in field-collected samples (not from bait trials) from 2015 and 2016 growing season

| Location | %T509 | CI %T509 | S509 copies µL^-1^ | CI S509 copies µL^-1^ | T509 copies µL^-1^ | CI T509 copies µL^-1^ |
| --- | --- | --- | --- | --- | --- | --- |
| **Western Australia 2015** |  |  |  |  |  |  |
| Bakers Hill 1 | 92.84 | 88.921 -- 96.796 | 104.36 | 99.364 -- 109.6 | 1353.7 | 1329.1 -- 1378.7 |
| Bakers Hill 2 | 100 | 91.041 -- 108.97 | 9.11E-02 | 1.28E-2 -- 0.647 | 3413.8 | 3329.1 -- 3500.6 |
| Bakers Hill 3 | 99.9 | 95.961 -- 103.94 | 0.714 | 0.395 -- 1.289 | 987.52 | 968 -- 1007.4 |
| Bakers Hill 4 | 100 | NA | 0 | NA | 942.56 | 921.89 -- 963.69 |
| Bakers Hill 5 | 99.97 | 95.982 -- 104.02 | 0.264 | 9.90E-2 -- 0.703 | 857.11 | 839.36 -- 875.24 |
| Bunbury 1 | 99.97 | 95.751 -- 104.27 | 0.24 | 7.74E-2 -- 0.744 | 708.03 | 691.28 -- 725.19 |
| Dandaragan 1 | 99.85 | 89.457 -- 111.38 | 6.12E-02 | 8.62E-3 -- 0.434 | 41.278 | 38.252 -- 44.543 |
| Dandaragan 2 | 97.96 | 87.427 -- 109.69 | 0.957 | 0.556 -- 1.648 | 45.958 | 42.463 -- 49.742 |
| Dandaragan 3 | 99.99 | 95.672 -- 104.37 | 8.81E-02 | 1.24E-2 -- 0.626 | 1164.6 | 1139.8 -- 1190 |
| Eaton 1 | 99.95 | 95.772 -- 104.17 | 0.719 | 0.374 -- 1.382 | 1343.5 | 1317.1 -- 1370.5 |
| Esperance 1 | 98.06 | 93.779 -- 102.45 | 8.531 | 7.198 -- 10.112 | 430.87 | 419.69 -- 442.34 |
| Esperance 2 | 100.00 | 95.16 -- 104.86 | 7.62E-02 | 1.07E-2 -- 0.541 | 2293 | 2251 -- 2335.9 |
| Frankland 1 | 99.87 | 93.614 -- 106.46 | 0.191 | 6.17E-2 -- 0.593 | 141.79 | 135.84 -- 148 |
| Frankland 2 | 99.98 | 95.441 -- 104.65 | 6.89E-02 | 9.71E-3 -- 0.489 | 412.29 | 401 -- 423.9 |
| Frankland 3 | 100.00 | NA | 0 | NA | 20.502 | 18.377 -- 22.872 |
| Frankland 4 | 99.68 | 84.893 -- 116.94 | 8.16E-02 | 1.15E-2 -- 0.58 | 25.137 | 22.468 -- 28.122 |
| Geographe 1 | 99.91 | 95.662 -- 104.23 | 0.753 | 0.392 -- 1.448 | 813.51 | 794.73 -- 832.74 |
| Kalannie 1 | 99.86 | 95.579 -- 104.22 | 0.969 | 0.55 -- 1.707 | 668.65 | 652.42 -- 685.28 |
| Kendenup 1 | 99.97 | 94.807 -- 105.35 | 6.32E-02 | 8.90E-3 -- 0.449 | 233.85 | 226.05 -- 241.92 |
| Kendenup 2 | 99.7 | 85.667 -- 115.98 | 6.34E-02 | 8.94E-3 -- 0.45 | 21.836 | 19.637 -- 24.281 |
| Kendenup 3 | 99.92 | 91.815 -- 108.66 | 6.30E-02 | 8.88E-3 -- 0.447 | 75.263 | 71.046 -- 79.729 |
| Kendenup 4 | 99.70 | 90.855 -- 109.31 | 0.232 | 7.49E-2 -- 0.72 | 76.849 | 72.137 -- 81.868 |
| Kojonup 1 | 99.72 | 85.808 -- 115.79 | 8.54E-02 | 1.20E-2 -- 0.607 | 30.243 | 27.235 -- 33.584 |
| Kojonup 2 | 99.91 | 95.196 -- 104.74 | 0.611 | 0.347 -- 1.075 | 624.48 | 612.47 -- 636.72 |
| Kojonup 3 | 98.94 | 86.778 -- 112.72 | 0.417 | 0.173 -- 1.001 | 38.98 | 35.579 -- 42.706 |
| Kojonup 4 | 99.80 | 91.028 -- 109.34 | 0.146 | 3.66E-2 -- 0.585 | 72.633 | 68.193 -- 77.361 |
| Kondindin 1 | 99.67 | 84.714 -- 117.17 | 6.89E-02 | 9.70E-3 -- 0.489 | 20.632 | 18.414 -- 23.117 |
| Kondindin 2 | 99.54 | 82.325 -- 120.27 | 7.67E-02 | 1.08E-2 -- 0.545 | 16.678 | 14.596 -- 19.057 |
| Mandurah 1 | 99.91 | 95.475 -- 104.44 | 0.605 | 0.289 -- 1.27 | 658.59 | 641.97 -- 675.64 |
| Mount Barker 1 | 99.92 | 95.802 -- 104.08 | 1.095 | 0.648 -- 1.848 | 1296.2 | 1270.8 -- 1322.2 |
| Mount Barker 2 | 99.85 | 94.695 -- 105.19 | 0.482 | 0.216 -- 1.072 | 313.51 | 303.24 -- 324.13 |
| Narrogin 1 | 100 | NA | 0 | NA | 147.3 | 140.54 -- 154.39 |
| Narrogin 2 | 99.23 | 90.634 -- 108.56 | 0.616 | 0.308 -- 1.231 | 79.264 | 74.5 -- 84.333 |
| Narrogin 3 | 65.76 | 61.007 -- 70.829 | 55.743 | 51.74 -- 60.055 | 107.08 | 101.42 -- 113.05 |
| Narrogin 4 | 99.61 | 89.756 -- 110.46 | 0.185 | 5.95E-2 -- 0.572 | 46.563 | 43.33 -- 50.037 |
| Perth 1 | 100 | NA | 0 | NA | 573.99 | 559.3 -- 589.07 |
| Perth 2 | 99.98 | 95.022 -- 105.09 | 9.20E-02 | 1.30E-2 -- 0.653 | 451.41 | 437.83 -- 465.42 |
| South Stirlings 1 | 98.17 | 93.771 -- 102.68 | 11.201 | 9.45 -- 13.276 | 601.26 | 585.8 -- 617.12 |
| South Stirlings 2 | 99.32 | 89.216 -- 110.46 | 0.454 | 0.189 -- 1.091 | 65.808 | 61.131 -- 70.843 |
| South Stirlings 3 | 99.83 | 95.292 -- 104.49 | 0.938 | 0.52 -- 1.694 | 558.66 | 543.77 -- 573.95 |
| South Stirlings 4 | 99.84 | 95.569 -- 104.2 | 1.156 | 0.685 -- 1.952 | 726.56 | 709.25 -- 744.29 |
| Wickerpin 1 | 98.97 | 87.855 -- 111.4 | 0.462 | 0.207 -- 1.027 | 44.293 | 40.787 -- 48.101 |
| Wickerpin 2 | 99.78 | 94.628 -- 105.1 | 0.87 | 0.468 -- 1.616 | 394.34 | 382 -- 407.07 |
| York 1 | 99.98 | 94.864 -- 105.12 | 0.46 | 0.219 -- 0.965 | 2219.8 | 2179 -- 2261.4 |
| **Queensland 2015** |  |  |  |  |  |  |
| Queensland 78 | 0.1 | 1.76E-2 -- 0.572 | 65.056 | 61.073 -- 69.298 | 6.57E-02 | 9.25E-3 -- 0.466 |
| Queensland 79 | 0.28 | 9.3E-2 -- 0.812 | 66.726 | 62.818 -- 70.877 | 0.184 | 5.95E-2 -- 0.572 |
| Queensland 80 | 3.96E-4 | 6.86E-3 -- 0.224 | 155.24 | 149.1 -- 161.63 | 6.14E-02 | 8.65E-3 -- 0.436 |
| Queensland 81 | 0 | NA | 32.605 | 29.842 -- 35.624 | 0 | NA |
| **Tasmania 2015** |  |  |  |  |  |  |
| Tasmania 1 | 99.93 | 95.67-- 104.26 | 0.59 |  | 849.48 |  |
| Tasmania 2 | 84.49 | 80.7-- 88.33 | 150.22 |  | 818.09 |  |
| **Tasmania 2016** |  |  |  |  |  |  |
| Tasmania 1 | 42.04 | 40.19 -- 43.932 | 925.78 | 906.05 -- 945.95 | 671.44 | 655.55 – 687.71 |
| Tasmania 2 | 57.21 | 54.036 – 60.393 | 1450.4 | 1421.7 – 1479.6 | 1938.9 | 1901.8 – 1976.7 |

CI = 95% confidence interval
